# Endurance Exercise Diverts the Balance between Th17 Cells and Regulatory T Cells

**DOI:** 10.1371/journal.pone.0074722

**Published:** 2013-10-09

**Authors:** Chava Perry, Marjorie Pick, Nir Bdolach, Inbal Hazan-Halevi, Sigi Kay, Idit Berr, Adi Reches, Yair Harishanu, Dan Grisaru

**Affiliations:** 1 Department of Hematology and Bone Marrow Transplantation, Tel Aviv Sourasky Medical Center, Tel Aviv, Israel; 2 Department of Hematology, Hadassah University Hospital, The Hebrew University, Jerusalem, Israel; 3 Gynecologic Oncology Unit, Department of Obstetrics and Gynecology, Lis Maternity Hospital, Tel Aviv Sourasky Medical Center, Tel Aviv, Israel; New York University, United States of America

## Abstract

Endurance, marathon-type exertion is known to induce adverse changes in the immune system. Increased airway hyper-responsiveness and airway inflammation are well documented in endurance athletes and endurance exercise is considered a major risk factor for asthma in elite athletes. Yet, the mechanisms underlying this phenomenon are still to be deduced. We studied the effect of strenuous endurance exercise (marathon and half-ironman triathlon) on CD4+ lymphocyte sub-populations and on the balance between effector and regulatory CD4+ lymphocytes in the peripheral blood of trained athletes, Endurance exercise induced a significant increase in Th17 cells and a sustained decrease in peripheral blood regulatory T cells (Tregs). While interleukin (IL)-2 levels remained undetectable, post-race serum IL-6 and transforming growth factor (TGF) β levels were significantly elevated. Treg levels in sedentary controls' decreased *in vitro* after incubation with athletes' post-exercise serum, an effect that was attenuated by supplements of IL-2 or anti IL-6 neutralizing antibodies. Our data suggest that exercise-induced changes in serum cytokine levels promote alterations in Tregs and Th17 cell populations, which may divert the subtle balance in the immune system towards inflammation. This may explain allergic and autoimmune phenomena previously reported in endurance athletes and contribute to our understanding of exercise-related asthma.

## Introduction

Accumulating clinical data support the notion that the immune system responds to increased physical activity. The reported effects of endurance exercise on the immune system differ from that of moderate physical activity. Moderate exercise is associated with reduced incidence of upper respiratory tract (URT) infections while endurance, elite athletes report of susceptibility to upper respiratory infections [1]–[4]. Endurance sport is considered a major risk factor for asthma. In fact, self-reported and physician-diagnosed asthma are twice as common in elite Norwegian and Finnish athletes than in randomly selected age-matched and sex-matched control populations [5], [6]. It was recently reported that the prevalence of allergy in a large cohort of runners competing in the 2010 London Marathon was 40% and almost half of the runners experienced upper respiratory tract (URT) symptoms [7]. Ceasing high-level training could lead to attenuation or even disappearance of bronchial hyper responsiveness and asthma [8].

The effector T-cell lineage shows great plasticity. T helper (h)17 cells are CD4+ lymphocytes that produce Interleukin (IL)-17, a cytokine that play a crucial role in allergic inflammation and are known as powerful pro-inflammatory cells that promote autoimmunity [9], [10]. On the other end of the spectrum CD4+CD25+ regulatory T cells (Tregs) are differentiated T lymphocytes actively involved in control of peripheral immunity. The identification of these cells has led to new insights into mechanisms of tolerance breakdown in human diseases, including those resulting from allergic, autoimmune, or infectious causes [11], [12].

The transcription factor Forkhead box P (FoxP)3 plays a key role in Tregs cell function and is a useful characteristic that allows identification for these cells. Transforming growth factor (TGF)β triggers FoxP3 expression in CD4+CD25- precursors and together with IL-2, is a key regulator of the signaling pathways that maintain FoxP3 expression and suppressive function in Tregs [13], [14]. However, a combined signal of TGFβ and the pro-inflammatory cytokine IL-6 may down regulate FoxP3 function, promote the induction of the transcription factor RAR-related orphan protein gamma (RORγ) and the differentiation of Tregs into Th17 cells [15], [16]. The balance between Th17 and Tregs CD4+ lymphocytes is tightly controlled as perturbed equilibrium might lead to decreased immune reactivity or, in contrast, to pathological inflammation [17].

We hypothesized that strenuous endurance exercise affects the balance between effector and regulatory CD4+ lymphocytes. As a result, we evaluated the peripheral blood CD4+ T cell sub-populations' profile in trained athletes throughout extreme exertion (marathon and half Ironman triathlon). Our data shows that endurance exercise induced a significant increase in Th17 cells and a sustained decline in peripheral blood Tregs population. Athletes' serum collected post-exercise induced, *in vitro*, a decrease in controls' peripheral blood Treg levels. These alterations in CD4+ T cell sub-populations may be attributed to changes in TGFβ, IL-6 and IL-2 serum levels, and perhaps, play a pivotal role in the mechanism(s) underlying allergic and autoimmune phenomena previously reported in endurance athletes.

## Materials and Methods

### Athletes

Forty two athletes who participated in the 2007 Emek-Hayarden Half Ironman triathlon (consisting of 1.9 kilometer (km) of swimming, 90 km of cycling and 21 km of running), or in the 2009 Tiberia marathon (a 42.2 km run), were included in this study. Athletes were included if they were over the age of 18 years old and up to 60 years old, with no previous known medical history of ischemic heart disease, hypertension or diabetes. Athletes with previous known medical history of ischemic heart disease, hypertension or diabetes were excluded from the study. This research was approved by the Tel Aviv Sourasky Medical Center Ethics Committee and all subjects participating in this research signed an informed consent form, approved by the Tel Aviv Sourasky Medical Center Ethics Committee.

All athletes filled a questionnaire documenting their age, gender, medical history, use of medications and any physical phenomena that occurred in the seven days following the race.

Blood samples were collected 4 days prior to the race (pre-race), at the end of the race (post-race) and 10 days after the race (recovery). Blood was drawn into EDTA tubes for complete blood count and flow cytometry immunophenotyping. Serum was separated from whole blood by centrifugation for 10 minutes at 2000 rpm and stored at −80°C until use.

Complete blood counts, including white blood cell sub-populations were analyzed with the Coulter LH 750 Analyzer (Beckman Coulter).

### Flow cytometry and intracellular staining of FoxP3 and IL-17

To detect Treg cells by flow cytometry we used a commercial kit containing anti-human CD4-PerCP, anti-human CD25-APC and cytoplasmic anti-human FoxP3-PE, according to the manufectorer instructions (eBioscience, CA, USA). Briefly, peripheral blood mononuclear cells (PBMC) were separated from whole blood on Ficoll-Hypaque (GE Healthcare Bio-Sciences, Uppsala, Sweden) and stained with antibodies aimed at surface CD4 (anti-human CD4-PerCP) and CD25 (anti-human CD25-APC). The cells were then fixed and permeabilized to detect expression of cytoplasmic FoxP3 (anti-human FoxP3-PE). Using four-color flow cytometry a lymphocyte population was first gated from the PBMCs according to their low forward versus side low scatter variables. Of the total lymphocyte gate only CD4^+^ cells were identified, gated and analyzed for their expression of CD25 and FoxP3. Isotype control IgG-PE and IgG-APC were used to detect background staining.

For the expression of Th17 on the cells, PBMC were either stimulated for 4 hours with 50 ng/ml phorbol esther myristate [PMA (Sigma)] and 250 ng/ml ionomycin (Calbiochem) in the presence of 2 µM Monesin, GolgiStop (eBioscience). To detect background or no stimulation, the cells were incubated in the presence of GolgiStop alone. The PBMC were stained with CD4-FITC (eBioscience) then fixed and permeabilized (Fix/Perm kit, eBioscience) and stained with anti IL-17 PerCP-Cy5 (eBiosciences). Using four-color flow cytometry a lymphocyte population was first gated from the mononuclear cell population according to their low forward versus side low scatter variables. Of the total lymphocyte gate only CD4^+^ cells were identified, gated and analyzed for their expression of IL-17. Isotype control IgG PerCp-Cy5 was used to detect background staining.

### Cytokines

TGF- β, IL-2, IL-6 and IL-10 serum levels were evaluated by ELISA, according to the manufacturer's instructions (Quantikines kits, R&D Systems). Tumor necrosis factor (TNF) α, interferon (INF) γ and IL-4 serum levels were evaluated by flow cytometry, using a particle-based immunoassay (CBA kit; BD Biosciences) according to the manufacturer instructions.

Serum cortisol levels were measured by electrochemiluminescent (ECL) method (Elecsys 2010 Immunoassay Analyzer, Boehringer, Mannheim, Germany).

### *In vitro* studies

PBMNC were separated from whole blood of healthy sedentary volunteers (males, median age 36 years, range 30–43), who were not regularly engaged in any sportive activity, on Ficoll-Hypaque gradients. 10×10^6^ cells were plated in 6 well plates and incubated for 4 hours in RPMI (Biological Industries, Beit Haemek, Israel), supplemented with 10% serum collected either pre-race or post-race from 4 of the participating athletes. One of the athletes' sera was used in 2 different sets of the *in vitro* experiments, with two different controls' PBMNC. Each serum was tested individually on individual control PBMNC.

Either IL-2 (50 U/ml), anti human IL-6 neutralizing antibodies (5 µg/ml) or mouse IgG isotype control antibodies (R&D Systems) were added to the post-race serum. Cells were then analyzed by flow cytometry for Treg cells, as described above.

The fraction of dead cells was evaluated by 7-amino-actinomycin D (7-AAD) staining (Sigma) for flow cytometry, according to the manufacturer instructions.

### Statistics

ANOVA was used to assess differences among the 3 time points. *p* value<0.05 was considered statistically significant.

Paired *t* test was used to assess differences between the effect of athletes' pre- and post-race serum on PBMNC from healthy sedentary controls.

## Results

Forty-two athletes were included in this study. Twenty-three athletes participated in the Half Ironman triathlon, of them 22 were males and one female; median age was 38 years (range 26–53). Twenty-two athletes finished the race. Nineteen athletes participated in the marathon [16 males and 3 females; median age 38 years (range 28–52)]. Sixteen athletes finished the race. Data regarding athletes who did not finish the exercise were not included in the post-race and recovery analysis.

All athletes filled a questionnaire documenting their medical history, use of medications and any physical phenomena that occurred in the seven days following the race. One athlete was using statins for hyper cholesterolemia. Eight athletes were using a multi-vitamin preparation, 7 athletes were using vitamin C supplements and 7 athletes were using magnezium supplements.

Three athletes reported that they suffer from asthma, three reported history of allergic rhinitis and one reported of allergy to mite.

When interviewed ten days after the race, athletes reported suffering from rhinitis (five athletes), clear sputum (one), dry cough (two), sore throat (one) and diarrhea (two), all within 72 hours after the race.

### Endurance exercise induced a transient decrease in CD4+ cell levels

Mean total white blood cell (WBC) increased significantly from 6.13±1.14×10^3^/µL pre- race to 16±3.72×10^3^/µL (p<0.001) immediately post-exercise, mostly due to increased neutrophil counts and returned to pre-race values in the recovery samples (Table 1). Post-race lymphocyte counts decreased only slightly and recovered 10 days later (1.72±0.56×10^6^/mL, 1.58±0.59×10^6^/mL, and 1.87±0.47×10^6^/mL in pre-race, post-race and recovery counts, respectively; *p* = 0.009 (Table 1)].

**Table 1:** Exercise-induced changes in white blood cell and CD4+ lymphocytes.

Mean±SD	Pre-race	Post-race	Recovery	*p* value
**WBC (×10^3^ µL)**	6.13**±**1.14	16**±**3.72	6.65**±**1.66	<0.001
**Neutrophils (×10^3^ µL)**	3.68±0.98	13.39±3.32	3.99±1.57	<0.001
**Lymphocytes (×10^3^ µL)**	1.72**±**0.56	1.58**±**0.59	1.87**±**0.47	<0.002
**% CD4^+^ lymphocytes**	24.28**±**7.2	5.45**±**3.36	26.49**±**8.23	<0.001
**% CD4^+^CD25^+^ in CD4^+^ lymphocytes**	9**±**4.9	9.14**±**6.41	8.83**±**7.4	NS
**% Tregs in CD4^+^ lymphocytes**	6.4**±**1.8	2.1**±1.3**	2.6**±1.4**	<0.001
**Absolute Treg counts (×10^4^/ml)**	17**±2**	2.7**±2**	5.7**±4**	0.002
**% Th17 cells in CD4^+^ lymphocytes**	1.16**±1.39**	2.96**±1.58**	1.07**±**0.68	0.002
**Absolute Th17 cell counts (×10^4^/ml)**	3**±4**	12**±6**	2**±1**	3×10^−5^

Shown are peripheral blood counts of white blood cells and differential counts of neutrophils, lymphocytes Coulter LH 750 Analyzer (Beckman Coulter) and percentage of CD4+, CD4+CD25+, FoxP3+Treg and Th17 lymphocytes (Flow cytometer) as measured 4 days before endurance exercise (pre-race), at the end of exercise (post-exercise) and 10 days after the competition (recovery). NS = not statistically significant.

The percentage of CD4+ lymphocytes decrease significantly from 24.28±7.2% in pre-race samples to 5.45±3.36 post-race (p<0.001; Table 1). Pre-race values returned to normal in the recovery samples.

### Th17 cell levels increased after strenuous endurance exercise

**Figure 1:**
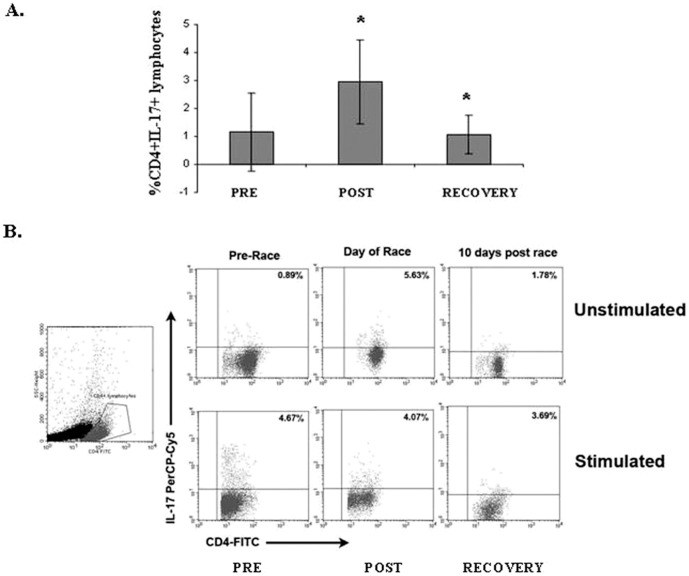
Th17 levels increase in response to strenuous endurance exercise. (**A**) Mean values of unstimulated Th17 cells, in response to endurance exercise. (**B**) Representative Th17 cell levels, as detected by flow cytometry: **Upper panel:** unstimulated Th17 cells (pre-stimulation with PMA was not used prior to detection of IL-17). Lower panel: levels of PMA-stimulated Th17 cells Pre = pre-exercise, Post = post-exercise, Recovery = 10 days post-exercise. * = statistically significant differences (p<0.05).

In order to study the effects of exercise on CD4+ lymphocyte sub-populations, we first studied changes in Th17 levels in response to strenuous endurance exercise. While in mice, Th17 cell levels can be evaluated from unstimulated CD4+ lymphocytes, measurements of Th17 cell levels in humans require *in vitro* stimulation of CD4+ lymphocytes. When the percentage of PMA-stimulated pre-race Th17 cells was compared to post-race levels, no significant differences were detected (3.06±1.76% vs. 3.18±1.19%, respectively, Figure 1). Surprisingly, when peripheral blood levels of Th17 were evaluated in unstimulated CD4+ lymphocytes, we detected a significant increase in both relative and absolute post-race Th17 levels, as compared to pre-race levels. Peripheral blood Th17 cell levels increased from 1.16±1.39% pre-race to 2.96±1.58% post-race, p = 0.002 (Figure 1). Absolute Th17 cell counts increased from 3±4×10^4^ cells/ml pre-race to 12±6×10^4^ cells/ml post race (p = 3×10^−5^). These data suggest that the effect of endurance exercise on Th17 levels can be detected without *in vitro* stimulation, and, perhaps, cannot be further expanded by in vitro stimulation.

### Strenuous endurance exercise induced a sustained decrease in peripheral blood Tregs

Next, we evaluated the effects of endurance exercise on the immune restraining Tregs. Although the percentage of CD4+ lymphocytes decreased, the percentage of activated CD4+CD25+ lymphocytes did not change in response to endurance exercise (Table 1).

**Figure 2:**
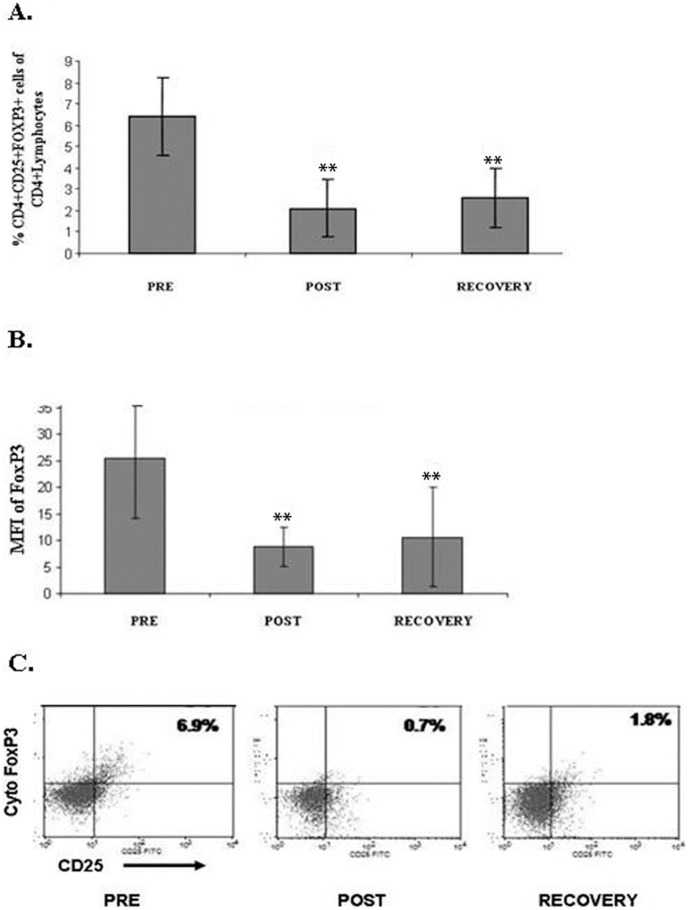
Sustained decrease in Tregs and FOXP3 levels in response to endurance exercise. Percent change in of CD4+CD25+FoxP3+ (Tregs), from the total CD4+ lymphocytes (**A**) and mean fluorescence intensity (MFI) of FOXP3 within the Tregs (**B**) in response to endurance exercise, as analyzed by flow cytometry. (**C**) Representative Tregs levels, as detected by flow cytometry. Pre = pre- exercise, Post = post- exercise, Recovery = 10 days post-exercise. ** = statistically significant differences (p<0.01).

Furthermore, the percentage of Tregs (CD4+CD25+FOXP3+ lymphocytes) decreased significantly post race and did not recover even 10 days later: Peripheral blood Treg levels were 6.4±1.8%, 2.1±1.3% and 2.62±1.4%, in pre-race, post-race and recovery samples, respectively, p<0.001; Figure 2A,C). Absolute Treg cell counts were 17±2×10^4^ cells/ml, 2.7±2×10^4^ cells/ml and 5.8±4×10^4^ cells/ml in pre-race, post-race and recovery samples, respectively, p = 0.002), suggesting that endurance exercise induces sustained suppression of peripheral blood (PB) Tregs population. Mean fluorescence intensity (MFI) of FoxP3 expression in CD4+CD25+ lymphocytes decreased significantly post exercise, as compared to pre-race measurements (from MFI of 25.38±11.28 to 8.8±3.73, p<0.0001). FoxP3 remained low, even after a 10 days recovery (MFI 10.65±9.32; (Figure 2B), suggesting that not only Treg lymphocytes decreased but also FoxP3 level per cell was affected by exercise.

### Endurance exercise induced a unique cytokine profile

**Figure 3:**
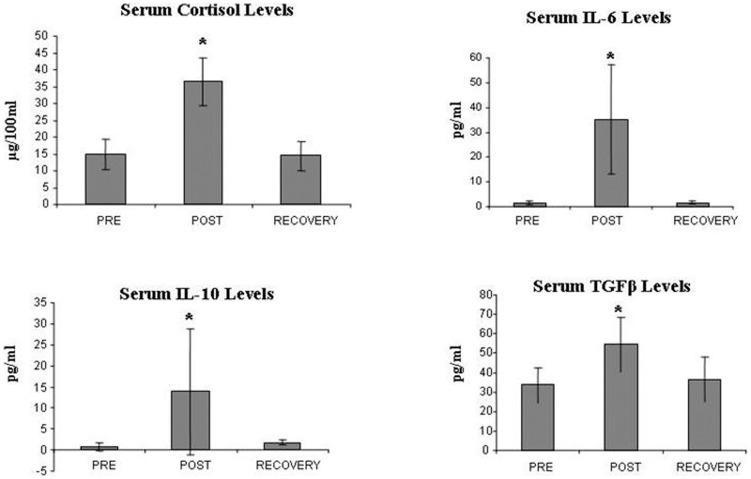
Endurance exercise induced changes in cytokine levels. Shown are changes in serum levels of cortisol, IL-6, IL-10 and TGFβ (ELISA) in response to endurance exercise. Pre = pre- exercise, Post = post- exercise, Recovery = 10 days post-exercise. * = statistically significant differences (p<0.05).

Serum levels of IL-2, IL-4, IL-6, IL-10, TNF-α, TGF-β, INF-γ and cortisol were studied in samples collected pre-race, post-race and at recovery. The levels of IL-2, IL-4, INF-γ and TNF-α were practically undetectable pre-race and did not change significantly in response to exercise. However, mean post-race levels of IL-6, IL-10, cortisol and TGF-β were significantly increased as compared to pre-race measurements and returned to baseline values after recovery (Figure 3): IL-6 increased from 1.56±0.66 pg/mL (pre-race) to 35.15±21.97 pg/ml (post-race, ANOVA p<0.05), a 28.69 fold increase. IL-10 increased from 0.81±1.01 pg/ml to 13.8±15 pg/ml (p<0.01). Cortisol levels increased from 14.86 ±4.66 µg/dL to 36.34±7.25 µg/dL (p<0.001and TGFβ levels increased from 33,418±8917 pg/ml to 54,355±14,050 (p<0.05).

### Post-race serum can induce a decrease in Treg levels *in vitro*

**Figure 4:**
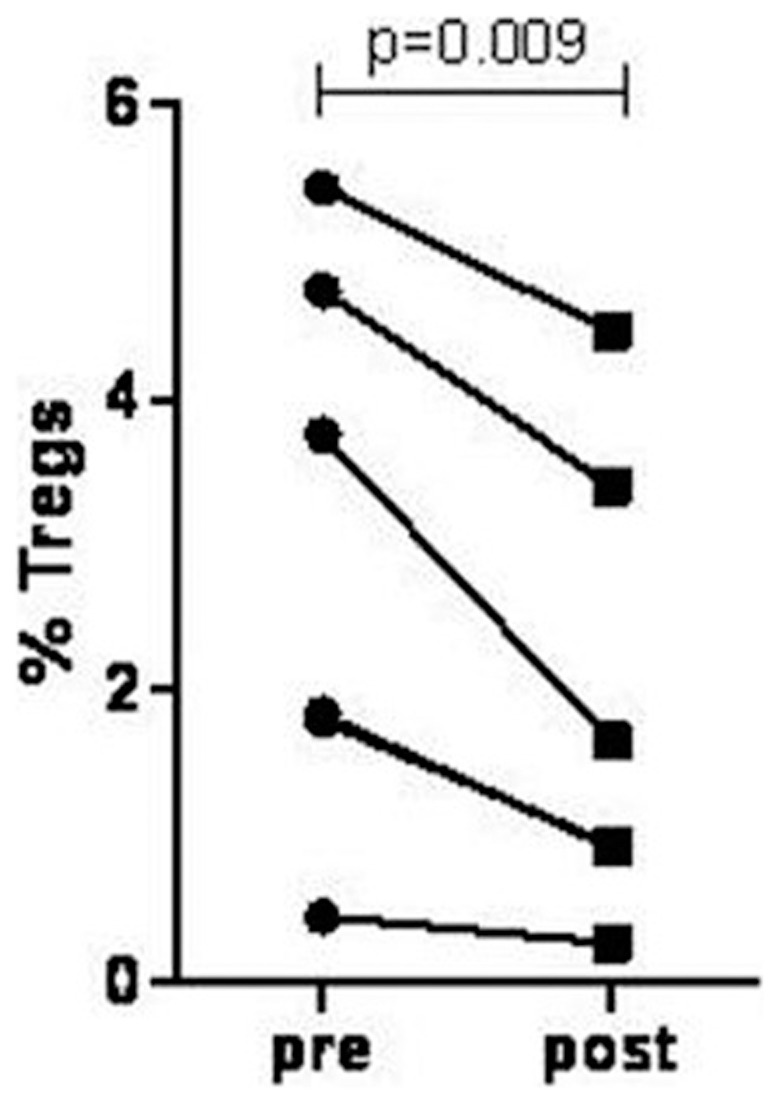
Post-race serum can induce a decrease in Tregs levels *in vitro*. Shown are changes in controls' PBMNC that were incubated for 4 hours with athletes' pre-race (pre) and post-race (post) serum. Athletes post-race serum induced a significant decrease in controls Tregs levels (mean percent of Tregs 3±1.93% and 1.9±1.6% in pre- and post-race serum, respectively; p = 0.009 Student *t* test).

PBMNC from healthy sedentary controls were incubated with athletes' serum collected pre- and post-race. Athlete's post-race serum induced a 35% decrease in Treg percentages in controls' PBMNC, as compared to cells incubated with athlete's pre-race serum or with control's own serum [(from 3±1.9% to 1.9±1.6%, p = 0.009; paired *t* test (Figure 4)]. CD4+ lymphocyte levels remained stable and unaffected by athletes' post-race serum. The fraction of dead or dying cells was less than 1%, as estimated by 7-AAD staining (data not shown), suggesting that the decrease in Treg levels could not be attributed to cell death.

Supplementing the cultures containing post-race serum with either IL-2 or anti IL-6 neutralizing antibodies, but not with IL-6 isotype control antibodies, minimized the decrease in Treg levels (from 3±1.9% to 2.33±1.93% and 2.2±2.2% with IL-2 and anti IL-6 antibodies, respectively), suggesting that changes in IL-6 and IL-2 levels may play a significant role in the observed exercise-induced alterations in CD4+ lymphocyte sub-populations.

## Discussion

Although moderate exercise is associated with long-term anti-inflammatory effect [18]–[20], endurance sport is considered a major risk factor for asthma and respiratory symptoms. [21]. The focus on respiratory infections in exercise has been encouraged by reports on the increased frequency of URT infections in elite endurance athletes after ultra-endurance exercise and during periods of intensive training. However, the evidence to support the presence of infections is inconclusive. This has led to the development of the “noninfectious” hypothesis, in which self-reported URT symptoms are related to exercise-induced inflammatory stimuli [22]. Supporting the “noninfectious” hypothesis, Robson-Ansley et al. reported recently that the prevalence of allergy in recreational marathon runners was 40%, similar to that in elite athletes and higher than that in the general population [7].

Here we report that prolonged endurance exercise induces a transient increase in the levels of the potent pro-inflammatory Th17 effectors cells, and a reciprocal sustained decrease in peripheral blood Treg levels, an effect that may contribute to our understanding of exercise-related URT symptoms.

### The effect of strenuous endurance exercise on Th17

Th17 has been identified only recently as a T cell helper lineage that regulates inflammation by producing distinct cytokines such as IL-17 (reviewed in [23]). Although Th17 play a crucial role in clearing extra-cellular pathogens, there is growing evidence that these cells also contribute to the pathology of a range of inflammatory diseases. Unlike studies in animal models, investigating Th17 cells in humans require stimulation of CD4+ lymphocytes with PMA in order to detect this specific T cell sub-population. Strenuous endurance exercise induced a significant increase in Th17 effector cells. The effect of endurance exercise, reported here, was intense and detected in un-stimulated Th17 cells. The fact that no significant changes were recorded between pre-race and post-race PMA-stimulated Th17 populations suggests that Th17 cells were intensely stimulated by factors induced during exercise and could not react to additional, artificial stimulation.

### The effect of strenuous endurance exercise on Tregs

Endurance exercise induced a decrease in both the percentage of Treg lymphocytes and the intensity of FoxP3 expression in CD4+CD25+ cells. The decrease observed immediately post-exercise may have contributed to the decrease in total CD4+ lymphocytes (although the percentage of Tregs was calculated out of the CD4+ lymphocyte population), however, 10 days later, although CD4+ lymphocyte population returned to pre-race levels, Tregs population was still significantly suppressed and showed only partial reconstitution. One possible explanation may lay in Tregs' sensitivity to extra-cellular ATP. Extra-cellular ATP functions as an indicator of tissue damage that activates the immune system, but can also inflict cell death, which Tregs seems to be extremely sensitive to [24]. Exercise is known to induce extra-cellular ATP [25] and the decrease in Tregs detected immediately after the exercise may be contributed to ATP-mediated cell death. However, as ATP is a short-lived agent, this cannot explain the sustained decrease in the Tregs population.

### The effect of endurance exercise on cytokine profile

As previously reported [26]–[28], we detected a significant increase in TGFβ and IL-6 levels immediately post exercise, however, IL-2 levels were undetectable pre- exercise and remained undetectable in post-race measurements. Perhaps by using more sensitive methods to measure serum IL-2, a post-race decrease could have been detected.

Athletes' serum collected post-exercise, induced a significant decrease in controls' Tregs, *in vitro*, without affecting cell viability, an effect that was diminished by either IL-2 or IL-6 neutralizing antibodies. These *in vitro* studies suggest that exercise-induced decrease in Tregs population was affected by the increase in IL-6 and the decrease in IL-2 serum levels, induced by heavy exertion and probably cannot be attributed to cell migration or cell death.

There is growing data reporting that Tregs and Th17 are developmentally linked and the same naïve T cell precursor pool that generates Treg cells is capable of generating IL-17-producing CD4+ T helper cells (Th17) [28], [29]. The differentiation of naïve T cells to Tregs is driven by TGF-β and IL-2, while Th17 cell generation is induced by IL-6 and TGFβ [28], [30]–[32], although additional cytokines (IL-21, IL-23) promote Th17 differentiation [33]. The specific cytokine profile detected post-exercise (increased IL-6 and TGFβ) raises the possibility that at least to some extent, exercise-induced changes in CD4+ lymphocyte sub-populations may be attributed to re-programming of Tregs into Th17 cells.

Intense marathon-like endurance exercise induces a decrease in the levels of pro-inflammatory cytokines (IL-1, IL-2, and TNFα) and simultaneously enhances the concentrations of IL-4, IL-6, IL-10 and TGF-ß [25]. The increase in plasma IL-6 concentration during exercise can be attributed entirely to release from contracting muscle fibers. Thus, alongside with its role in promoting Th17 cells differentiation, during prolonged exercise muscle-derived IL-6 also promotes elevated secretion of cortisol and can induce the anti-inflammatory cytokines IL-1ra and IL-10 as well as supresses the pro-inflammatory TNFα. These anti-inflammatory changes probably aim at balancing and suppressing the ability of the immune system to inflict tissue damage [34].

Studies measuring the effect of exercise on Tregs in humans are scarce. Non-exhaustive exercise such as Thai Chi was associated with increased Treg levels, which coincides with the previously reported anti-inflammatory effects of moderate exercise [35]. On the other hand, a recent report suggested that moderate exercise had no effect on murine Tregs while intensive exercise induced increased Treg levels [36]. As the murine immune system differs from the human system, it is possible that in humans, moderate exercise is associated with an anti inflammatory CD4+ profile while intense, strenuous exercise is associated with a pro-inflammatory CD4+ populations.

**Figure 5:**
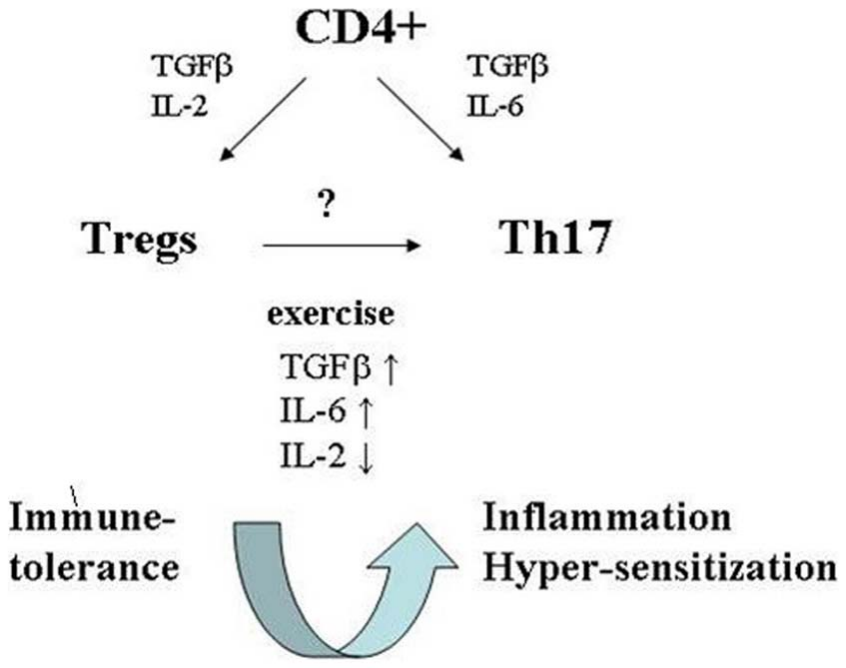
A model of homeostatic balance between Tregs and Th17 under endurance exercise. Endurance exercise induced a significant increase in Th17 cells and a sustained decline in peripheral blood Tregs population. These alterations in CD4+ T cell sub-populations may be attributed to changes in TGFβ, IL-6 and IL-2 serum levels.

In conclusion, increased Th17 cell levels, along with a protracted decrease in the levels of immune tolerance-inducing Tregs, may attenuate the subtle balance between immune tolerance and inflammation (Figure 5). This may explain, at least partially, allergic and autoimmune phenomena previously reported in association with strenuous endurance exercise and support the alternative, “non infectious” hypothesis, reasoning the self reported upper respiratory symptoms documented in endurance athletes [3], [37].
